# Amygdalar involvement in respiratory dysfunction

**DOI:** 10.3389/fphys.2024.1424889

**Published:** 2024-08-28

**Authors:** Pedro Trevizan-Baú, John A. Hayes, Donald C. Bolser, Leah R. Reznikov

**Affiliations:** Department of Physiological Sciences, University of Florida, Gainesville, FL, United States

**Keywords:** airway protection, cough, amygdala, anxiety, respiration

## Abstract

The brainstem has long been recognized as the major respiratory control center, but it has become increasingly appreciated that areas upstream of the brainstem modulate respiration and airway defensive behaviors. This review aims to define the role of the amygdala, a key temporal brain region essential for limbic function, in respiration and airway defenses. We summarize literature describing roles for the amygdala in control of respiration, swallow, cough, airway smooth muscle contraction, and mucus secretion. We emphasize the need to understand how the amygdala regulates these functions both at a local scale and network scale and identify knowledge gaps for current and future investigations. Lastly, we highlight literature suggesting that amygdala dysfunction may contribute to respiratory dysfunction.

## Introduction

Respiration is essential for the survival of all mammals. It is a vital motor function that controls respiratory muscles contributing to pulmonary oxygen uptake and excretion of carbon dioxides. Airway protective behaviors, such as cough and swallow, are also important because they prevent the aspiration of pathogen and particles to ensure patency and health of the airway for optimal gas exchange.

In the central nervous system, the major respiratory control center is in the brainstem, the distal (caudal) part of the brain. The brainstem respiratory network includes several brain regions in the medulla oblongata, such as the pre-Bötzinger (pre-BötC), Bötzinger (BötC), raphé, rostral and caudal subdivisions of the ventral respiratory groups (rVRG and cVRG, respectively), and the nucleus of the tractus solitarius (NTS); as well as nuclei in the pons, such as the Kölliker-Fuse (KF) and parabrachial nuclei. Over the last hundred years (Lumsden, 1923) until more recent days (Smith et al., 1991; Del et al., 2018; St-John and Paton, 2000; Dhingra et al., 2020; Dhingra et al., 2019), it has been widely demonstrated that respiration in mammals is generated and regulated within the ponto-medullary respiratory network. Additionally, the brainstem network drives orofacial motor behaviors, including airway protective behaviors such as cough and swallowing, that are coordinated with breathing (Moore et al., 2014). However, it has become increasingly appreciated that areas upstream of the brainstem modulate not only breathing, but also orofacial motor behaviors.

**FIGURE 1:**
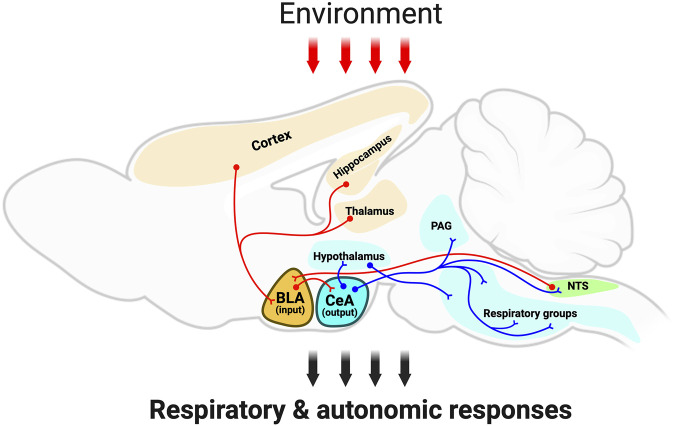
Schematic outlining the amygdala integratory function and neuronal anatomical connectivity. The schematic, red-colored neurons depict the sensory information from several brain regions, such as brainstem (i.e., NTS), cortex, thalamus, and hippocampus to the amygdala; and the red arrows depict sensory information from the environment targeting the amygdalar-neuroanatomical network. Note that the basolateral amygdala subdivision is a gating center that receives sensory information, which is further transmitted to the central amygdala. Then, the central amygdala sends projection neurons (blue-colored neurons) to hypothalamic centers, periaqueductal grey, as well as brainstem centers. This amygdalar-neuroanatomical framework might modulate the ongoing respiratory motor activity for respiration and airway-protective behaviors (e.g., cough, sneeze, laryngeal adduction, and swallow). Abbreviations: BLA, basolateral amygdala; CeA, central amygdala; Hypo, hypothalamus; PAG, periaqueductal gray; NTS, nucleus of the tractus solitarius; RG, respiratory groups (in the brainstem). Created with BioRender.com.

In the present review article, we highlight the amygdala as a key brain region that influences respiration and airway protective behaviors (Figure 1). The amygdala is of increasing interest in the airway biology field as it modulates autonomic responses to fearful stimuli, in part through activation of the sympathetic nervous system. Moreover, anxiety, which is in large part associated with exaggerated amygdala activity, is common in airway disease and exacerbates lung pathology. Here, we provide a summary of literature highlighting the amygdala in the control of respiration, cough, swallowing, airway smooth muscle contraction, and mucus secretion. Then, we discuss the amygdala in respiratory dysfunction and how it may be impacted in neurological diseases. Lastly, we identify knowledge gaps in the field and propose key questions for current and future investigations.

## Amygdala function and connectivity

The amygdala is an almond-shaped structure bilaterally located in the temporal lobes of the brain and is essential for detecting threats (Onat and Buchel, 2015; Rajbhandari et al., 2016; Kim et al., 2016). It receives sensory information from several brain regions, such as brainstem, cortex, thalamus and hippocampus. These inputs allow the amygdala to integrate sensory information, including taste, visceral, vision, audition, somatic sensation, and olfaction (Price, 2003).

Structurally, the amygdala is divided into multiple subdivisions. The basolateral amygdala (BLA) subdivision is a key gating center that receives sensory information from the environment and transmits it to the central amygdala (CeA) via excitatory glutamatergic pyramidal projection neurons (Reznikov et al., 2008). The central amygdala, in turn, sends projections to hypothalamic centers (Keifer et al., 2015) and periaqueductal grey (PAG) in order to mount the proper response to threats (Tovote et al., 2016). It is the CeA projections to the hypothalamic centers and to the NTS and rostral ventrolateral medulla in the brainstem that are critical for regulating autonomic and respiratory responses (Farkas et al., 1997; Chiou et al., 2014; Saha et al., 2005). Remarkably, it has been demonstrated that CeA projections to the midbrain PAG connect with medullary pre-motor neuronal targets to control (or integrate) specific evolutionary conserved defensive behaviors, such as freezing (Tovote et al., 2016).

It has been postulated that this simplified view of the amygdala having a main input center (basolateral amygdala, BLA) and output center (central amygdala, CeA) is relatively consistent across species. Such neural framework has been mainly elucidated in the mouse; however, experimental studies performed in other mammalian species, including primate and non-primates, suggest that the neuronal organization of amygdalar connections (i.e., afferences and efferences) may differ across species (McDonald, 1998). This may be evident by various ways that species display fear and emotional aspects as well as social behaviors. For instance, it is known that the neuroanatomical complexity of the amygdala in humans is greater, with six major subdivisions reported (Zhang et al., 2023), and the function of these subdivisions is still debated and based largely on cytoarchitecture. Nevertheless, it has been accepted that even non-mammalian species, including fish, reptile and birds, present an amygdalar brain circuit similar to the mammal amygdala, suggesting that amygdalar neuronal circuit is relatively conserved throughout evolution in vertebrates (Jarvis et al., 2005; Lanuza et al., 1998; Johnston, 1924; Ariëns Kappers et al., 1936; Janak and Tye, 2015).

Although the neuroanatomical connectivity of the amygdala within the brain as well as their function are still to be fully discovered, it is plausible to think that the neuroanatomical framework of the amygdala with widespread and diverse brain regions makes the amygdala a key subcortical region for processing contrasting and varied emotional behaviors, such as fearful and rewarding environmental stimuli. Overall, it has been suggested that distinct amygdala circuits may contribute to a wide array of behaviors (Swanson and Petrovich, 1998). For example, it is known from human and rodent experimental studies that *i)* the amygdala is activated by fear-conditioned stimuli and *ii)* anxiety-related behaviors strongly correlates with BLA and CeA neuronal activity (Janak and Tye, 2015). Not surprisingly, amygdalar-related behaviors are also correlated with respiration and autonomic responses. It is still unknown, however, whether this is a consequence of the amygdalar-related behaviors in autonomic function that may change respiration or whether the amygdala projection neurons targeting the brainstem respiratory centers are regulating respiratory (and/or autonomic) activity itself during different behaviors. The next section of this review discusses the functional role of the amygdala in the regulation of breathing.

## Functional evidence for amygdalar regulation of respiration

The amygdala has long been recognized to influence cardiovascular and respiratory responses (Kreibig, 2010; Masaoka and Homma, 2001). Thus, it is not surprising that the interest in the amygdala as modulator of respiratory function dates back several decades. In 1972, work in anesthetized cats described two major outcomes on respiration that were amygdala subdivision-dependent: respiratory depression and respiratory activation (Bonvallet and Bobo, 1972). The authors speculated that the increase in respiration that was accompanied by cardio acceleration was due to increases in sympathetic tone. Conversely, they speculated that the decrease in respiration that was associated with cardio deceleration was due to decreased sympathetic tone and enhanced parasympathetic tone. In 1983, another study demonstrated that low level stimulation of the CeA in awake rabbits induced bradycardia accompanied by an increase in respiration (Applegate et al., 1983). In the following year, another study demonstrated that high frequency stimulation (100 Hz) of the CeA in cats induced a large and sustained inspiratory effort (Harper et al., 1984). A few years later, another study in cats demonstrated that 22% of the CeA neurons discharged phasically with the respiratory cycle during at least one wake or sleep cycle (Zhang et al., 1986). Additional studies in experimental models of either awake or anesthetized rodents and rabbits supported amygdala subregion-dependent influence on respiration, suggesting an association between amygdala-related emotional responses and breathing (Nie and Liu, 1992; Sugita et al., 2015; Adamyan and Akopyan, 2006).

In humans, there have also been reports of increased or decreased respiratory effects due to activation or engagement of the amygdala. For example, *i*) electric stimulation of the amygdala increased respiratory rate in epileptic patients (Masaoka and Homma, 2004); and *ii*) lesions of the amygdala resulted in a reduction of respiratory rate during anticipatory anxiety (Masaoka et al., 2003). Other laboratories reported breathing dysfunction or apnea when seizures spread to the amygdala, as well as upon amygdala electrical stimulation (Dlouhy et al., 2015; Nobis et al., 2018; Nobis et al., 2019). These divergent effects of amygdalar regulation on respiration (either direct through stimulation or presumed through anxiety or fear-evoking events) have been further studied and reported in humans (Inman et al., 2020; Gomez et al., 2004; Boiten, 1998; Rhone et al., 2020). Additionally, psychological and clinical reports point toward the notion of a clear association between amygdala-related behaviors, such as panic attack, and hyperventilation (Thyer et al., 1984; Nardi et al., 2009). For example, Thyer and colleagues (1984) documented that acute hyperventilation can result in experienced levels of anxiety and tachycardia, suggesting that hyperventilation can lead to pathologic anxiety (Thyer et al., 1984). Taken together, functional data not only suggest that respiration is influenced by amygdala neuronal activity, but respiratory activity may also influence amygdala-related responses.

Few years later, such functional data in the literature became the basis for neuroanatomical studies attempting to investigate the neural connectivity between the amygdala and the key respiratory nuclei, located in the brainstem. Consistent with this, comprehensive neuroanatomical tracing experiments have shown a large number of neuronal projections from the amygdala to key respiratory control areas in the midbrain, pons and medulla (Yang et al., 2020; Trevizan-Baú et al., 2021a). As expected, the majority of the amygdala projections were found to target the midbrain PAG (Trevizan-Baú et al., 2021a). Similar observation of the amygdala-PAG neuroanatomical connectivity has been reported by others (Morrell et al., 1981; Mantyh, 1982; Rizvi et al., 1991). Then, the midbrain PAG further connects with the key respiratory nuclei in the ponto-medullary brainstem network (Inman et al., 2020). Remarkably, the midbrain PAG has been shown to modulate respiratory motor pattern (Subramanian, 2013; Farmer et al., 2014; Subramanian et al., 2008; Subramanian and Holstege, 2013; Hayward et al., 2003), particularly in the context of respiratory-related activity during defensive behavior (Hayward et al., 2003), which is also an amygdala-related emotional component. Therefore, it is possible that the amygdala-PAG connectivity plays crucial roles in modifying (modulating) the respiratory motor pattern via the PAG connectivity with the respiratory control areas in the pons and medulla (Trevizan-Baú et al., 2021b).

However, it is also important to note that amygdala neurons project to some of the ponto-medullary respiratory nuclei, bypassing the PAG (Yang et al., 2020; Trevizan-Baú et al., 2021a; Trevizan-Baú et al., 2024). These amygdalar inputs target some key respiratory nuclei such as the pontine KF and the medullary pre-BötC, BötC, and raphé regions. Although the neurochemistry of those connections is still to be discovered, it has been shown in the mouse that amygdalar monosynaptic projections to the medulla target both excitatory and inhibitory pre-BötC neurons (Nobis et al., 2018). Future studies addressing the neurotransmitter and/or receptor types of the amygdala connectivity with the respiratory neurons may shed light onto the neuronal mechanisms by which amygdala influence breathing. Overall, because respiration engages a distributed brainstem network (Dhingra et al., 2020; Dhingra et al., 2019), amygdala functional coupling with respiration might be explained by amygdalar neuroanatomical connectivity with all the key respiratory control areas within the brainstem, including in the midbrain PAG.

It is important to note that the aforementioned neuroanatomical studies investigated the amygdalar efferent projections to the brainstem network. As highlighted earlier, though, the amygdala also connects with cortical sensory systems, as well as the thalamus and hypothalamus (Figure 1) (Gouveia et al., 2019; Krohn et al., 2023), forming a large-scale brain network. Indeed, studies in humans have shown that breathing can be disrupted through a large limbic/paralimbic mesial temporal network that includes the amygdala (Lacuey et al., 2019). In these studies, stimulation of the hippocampus, amygdala, hippocampal gyrus and antero-mesial fusiform gyrus caused central apnea.

Consistent with this, experiments in anesthetized mice showed that ablation of orexin hypothalamic neurons profoundly impacted amygdala-induced cardiorespiratory responses, supporting that hypothalamus is also part of the amygdala-respiratory brain network (Zhang et al., 2009). When provided an auditory stimulus, rats also exhibit an increase in respiratory rate that was proportional to the intensity of the auditory stimulus (Bondarenko et al., 2014). Pharmacologic inhibition of the amygdala blunted the increase in respiration at the higher intensity auditory stimuli. The authors speculated that auditory effects on respiration were due to CeA projections to the dorsomedial hypothalamus (Figure 1) (Bondarenko et al., 2014). Combined, these findings highlight that the amygdala is a part of a network that modulates respiration, and it is the network response that dictates whether respiration is increased or decreased in response to amygdala activation or engagement.

## The role of the amygdala on airway protection

The primary and vital role of respiration is gas exchange. However, the respiratory system consists of fundamental reflex and non-reflex mechanisms (i.e., airway protective behaviors) to prevent pathogen and particles from reaching the lungs, which is essential for optimal gas exchange (for review, see Pitts, 2014) (Pitts, 2014). Airway protection is achieved through the activation of several potent reflexes mediated primarily by brainstem pathways (for review, see Bolser et al., 2015) (Bolser et al., 2015). These reflexes include cough, sneeze, laryngeal adduction and swallow. Collectively, these airway protective behaviors eject and/or prevent intrusion of foreign material into the subglottic airways and, thereby, reduce obstruction and the probability of pulmonary infection and inflammation. Except for the laryngeal adductor reflex, a significant amount of information exists regarding the neurogenesis of these behaviors. However, this body of knowledge is largely focused on brainstem regulation and circuits (Moore et al., 2014). The role of amygdala in controlling the expression of airway protective behaviors is still poorly understood.

Components of swallowing have a role in airway protection. Laryngeal elevation allows the epiglottis to move over the laryngeal orifice and protect it from intrusion of ingested food and liquids (Jean, 2001). Further, strong laryngeal adduction closes the airway and prevents aspiration of ingested material or saliva during swallowing (Jean, 2001). Electrical stimulation of the anterior amygdala and CeA enhanced the frequency of swallowing that was induced by ipsilateral electrical stimulation of the superior laryngeal nerve (Weerasuriya et al., 1979; Bieger et al., 1977; Bieger and Hockman, 1976). Lesioning experiments suggested that the faciliatory effect on swallowing was mediated via pathways involving the ansa peduncularis and median forebrain bundle (Weerasuriya et al., 1979). Microinjections of dopamine into the region the amygdala also facilitated reflexive swallowing (Weerasuriya et al., 1979), however the injection volumes were large (µL range). These results support a modulatory role of the amygdala in the production of swallowing.

There is no published evidence regarding the role of the amygdala in the production of the laryngeal adductor reflex (LAR). The LAR is a brief closure of the vocal folds that is activated by mechanical or electrical stimulation of laryngeal sensory afferents (Ludlow, 2005). It has an important role in preventing aspiration of material that enters the laryngeal vestibule (Ludlow, 2005). There are descending pathways from the amygdala to laryngeal adductor motoneurons (Arita et al., 1995; Simonyan and Jürgens, 2005; Van Daele and Cassell, 2009), which provide an anatomical substrate for laryngeal adduction.

The amygdala (e.g., CeA) also receives specific afferent input from tracheal sensory afferents that synapse in the NTS and other suprapontine brain regions (McGovern et al., 2012a; McGovern et al., 2012b), suggesting that the amygdala is part of the integrative brain circuit for sensations arising from the airways. This finding supports the concept that at least some of this afferent input is likely related to the production of airway reflexes, such as coughing. For example, electrical stimulation of the amygdala at low frequencies (5–10 Hz) will produce intense repetitive behaviors in the anesthetized cat that resemble coughing which they termed “spasmotic expiratory responses” (SER). However, based on the methods that were available the group that reported this finding (Kito et al., 1977a), it was not clear that these behaviors had all of the motor components of naturally induced coughing, such as laryngeal adduction, which is responsible for the compression phase of this behavior.

The duration of action of intravenous administration of the cough suppressant drugs, codeine and dextromethorphan, on the SER was less than on cough induced by electrical stimulation of the superior laryngeal nerve or mechanical stimulation of the trachea (Kito et al., 1977a). In other experiments, microinjection of codeine or dextromethorphan into the NTS had a greater suppressive effect on mechanically-induced coughs than the SER (Kito et al., 1977b). These observations suggest that circuits in the amygdala interact with the brainstem cough circuits in a manner that is less dependent on antitussive-sensitive elements than for induction of coughing by lower airway afferents. Coughing induced by mechanical stimulation of the larynx is less sensitive to antitussives than cough produced by tracheobronchial afferents (Korpas and Tomori, 1979). This observation raises the hypothesis that the SER from the amygdala may actuate brainstem circuits that are more related to the production of laryngeal than tracheobronchial coughing. The pathways by which circuits in the amygdala influence brainstem circuits include the stria terminalis (Kito et al., 1977b).

The amygdala itself mediates input from several areas of the limbic cortex to induce the SER and coughing. The SER was mainly depressed by electrical stimulation of cingulate, ectosylvian, and orbital cortical locations (Kasé et al., 1984). Similar but lower magnitude effects were observed on cough induced by electrical stimulation of the superior laryngeal nerve. Facilitation of the SER, mostly in the form of lowered threshold for activation, occurred in response to electrical stimulation of the piriform and olfactory cortices. There were some differential effects on the SER and peripherally induced coughing. Electrical stimulation of the suprasylvian cortex had no effect on the SER but enhanced peripherally induced cough. While electrical stimulation of the olfactory and piriform cortices enhanced the SER, those areas had no effect on peripherally-induced cough (Kasé et al., 1984).

Therefore, it is evident that the amygdala plays pivotal roles in modulating cough. However, it is also obvious that cough is coordinated by a large-scale brain network that is formed by neuroanatomical and functional connections between the amygdala and other brain regions such as the cortex, hypothalamus and the brainstem. As aforementioned, it is known that the amygdala projects (via monosynaptic inputs) to the respiratory control areas in the brainstem (Yang et al., 2020; Trevizan-Baú et al., 2021a). For instance, the pontine KF nucleus, which receives strong anatomical inputs from the amygdala, plays pivotal role in laryngeal adduction during orofacial behaviors, including swallowing (Bautista and Dutschmann, 2014; Dutschmann et al., 2014), and, thus, protecting the lower airways (Medda et al., 2003). Nevertheless, future studies need to address the functional role of amygdala-related neuroanatomical pathways on laryngeal adduction during airway protective behaviors.

## The role of the amygdala on airway smooth muscle and mucus production

Airway smooth muscle contraction and mucus secretion are key airway protective behaviors that facilitate airway patency by keeping foreign bodies and particles out of the lungs. Our understanding of the role of the amygdala in modulating these two vital defenses is limited. Some evidence suggests that amygdala activity in an experimental rodent model is associated with the regulation of airway smooth muscle constriction (bronchoconstriction) (Chen et al., 2020). In that study, lesions of the central amygdala decreased airway resistance, a proxy of smooth muscle relaxation directly related to the caliber, in healthy rats (Chen et al., 2020). Human adults with asthma show marked increases in airway resistance in response to fear (Smith et al., 1970). Similar findings have been reported in children with asthma when recalling fear, such that forced expiratory volume decreases during fear recall (Tal and Miklich, 1976), suggested increased airway resistance. Though the studies in humans are indirect evidence of amygdalar control of airway smooth muscle, a pathway where by the amygdala can modulate preganglionic motor neurons of the vagus nerve, has been reported in rodents, in the context of gut-related neurons in the dorsal vagal complex (Zhang et al., 2003). Because it is well known that dysregulation of parasympathetic nerves increases smooth muscle tone (i.e., bronchoconstriction) (Undem and Kollarik, 2005), future functional studies would be essential to test the hypothesis that the vagus nerve may also modulate the airway smooth muscle tone.

Given that the amygdala is a key regulator of the autonomic nervous system, it is possible that it also modulates airway mucus secretion from the glands throughout the airway tree. Though there are no direct studies that have investigated the role of the amygdala in mucus secretion throughout the airway, it is well documented that dysregulation of parasympathetic nerves contributes to excessive mucus production (Undem and Kollarik, 2005). Additionally, airway-innervating sympathetic neurons innervate blood vessels and submucosal glands of the bronchi (Oh et al., 2006) and one of the main triggers of sympathetic neural activity is the amygdala. Thus, future studies focused on amygdalar control of airway mucus secretion are warranted.

## Amygdala dysfunction in airway disease and respiratory dysfunction

Anxiety is a feeling of worry, fear, or unease that produces both physical and emotional symptoms. Anxiety is common among multiple airway diseases and associated with worsened airway pathology. For example, anxiety is reported in 7%–50% of people with chronic obstructive pulmonary disease (COPD) (Pumar et al., 2014) and increases hospitalizations and mortality (Divo et al., 2012; Tsiligianni et al., 2011). Anxiety often appears together with dyspnea in patients with COPD (Strang et al., 2014) and has been reported as a marker of acute exacerbation (Costi et al., 2006).

Similarly, it has been reported that anxiety and depression affect more than 35% of people with cystic fibrosis (CF) in the United States (Baiardini et al., 2015a). Globally, anxiety prevalence in people with CF is estimated to be 25% (Guta et al., 2021). On average these rates are approximately double what is observed in the general population (Lord et al., 2023). Anxiety and depression in people with CF are linked to poorer medical outcomes (Snell et al., 2014). For example, anxiety was associated with severity of chest symptoms in people with CF, whereas depression was associated with low lung function (Yohannes et al., 2012). Another study found that people with CF and anxiety reported more respiratory symptoms, such as difficulty in breathing (Havermans et al., 2008).

Anxiety is found in 25%–40% of asthma patients (McDonald et al., 2019; Sweeney et al., 2016), can precipitate asthma attacks (Urrutia et al., 2012), and is associated with greater frequency of exacerbations, poorer asthma control, and increased use of healthcare resources (Sastre et al., 2018). Chronic psychiatric illness, including anxiety, is a known risk factor for death from asthma (Fuhlbrigge et al., 2012; Sturdy et al., 2002). Similarly, individuals with severe asthma have a greater incidence of anxiety and depression compared to those with mild to moderate asthma (Baiardini et al., 2015b). Finally, some studies suggest that anxiety is a prodromal sign of an asthma exacerbation (Beer et al., 1987). The brain-lung mechanisms responsible for increased anxiety in lung disease are unknown; however, enhanced amygdala activity is one of the most consistent findings among people with anxiety and anxiety traits (Stein et al., 2007; Greenberg et al., 2017).

How anxiety worsens airway pathology is not entirely understood, though several clinical studies have shown that stress is associated with enhanced airway inflammation in asthmatic individuals (Wenzel, 2012; Trueba and Ritz, 2013; Rosenkranz et al., 2016; Rosenkranz et al., 2012; Marin et al., 2009; Loerbroks et al., 2014). Consistent with this, high levels of chronic stress elevate risk of asthma exacerbation up to 3 fold (Sandberg et al., 2000). Individuals with COPD and having high stress also exhibit greater systemic inflammation at baseline compared to those without high levels of stress (Gueli et al., 2011). It is well known that stress modifies amygdala activity (Reznikov et al., 2008; Reznikov et al., 2007). Thus, these studies serve as additional support that amygdala dysfunction may contribute to airway dysfunction. Alternatively, inflamed airways may also influence higher brain regions via ‘bottom-up’ pathways (from the lungs to the brain). Experimentally, this is supported by studies showing that allergic inflammation in the airways may trigger anxiety-like behavior by inducing structural and functional alteration in the brain, including the amygdala (Dehdar et al., 2019; Gholami-Mahtaj et al., 2022).

Another growing area of interest is the role of the amygdala in seizure-induced inhibition of respiration (Rhone et al., 2020). Some people with epilepsy experience sudden unexpected death in epilepsy (SUDEP) and cessation of respiration and/or hypoventilation are thought to contribute to SUDEP (Ryvlin et al., 2013; Bateman et al., 2010). In pediatric patients with seizures, apnea coincided with seizure spread to the amygdala (Rhone et al., 2020). The authors of this study further examined the role of the amygdala through direct stimulation and observed apnea in all patients when the amygdala was electrically stimulated. Electrical stimulation of adjacent brain regions or those that are not part of the amygdala network failed to elicit apnea. This selectivity of the amygdala contrasted with work performed by another group, who demonstrated that limbic/paralimbic network was responsible for central apnea (Lacuey et al., 2019). These divergent findings further emphasize the need to understand how and under what conditions the amygdala at the local and network scale regulate respiration.

Some clinical reports showed that breathing and associated autonomic functions (*e.g.*, arousal) are impacted by amygdala destruction (amygdalotomy). This surgical procedure has been used over the last decades attempting to reduce severe (intractable) aggressive behavioral disorders in some patients. Clinically, it has been observed that amygdalotomy leads to a decline in the level of autonomic arousal (Lee et al., 1998). However, it is unknown whether this is a consequence of the diminished aggressive behavior (i.e., indirect effect on breathing) or a direct effect of the neuroanatomical destruction on the neural control of breathing and autonomic function. It is important to note though that clinical reports of patients that underwent amygdalotomy are not consistent when comparing different clinical cases.

## Unresolved questions and topics for future investigation

The role of the amygdala in respiration appears to be more developed compared to our understanding of the amygdala in airway defensive behaviors. Therefore, research efforts focused on the amygdala in airway defense under healthy and diseases conditions could close this gap. There is no published information on the role of the amygdala in the production of the laryngeal adductor reflex, a behavior that minimizes intrusion of material into the upper trachea. However, the amygdalar pathways do project to laryngeal motoneurons (Arita et al., 1995; Simonyan and Jürgens, 2005; Van Daele and Cassell, 2009). Additionally, it is not clear whether there are amygdala subdivision-dependent effects on cough or airway smooth muscle contraction or mucus secretion like that observed in breathing.

Some of our insights into the relationship between the amygdala and respiration and defensive behaviors originate from studies associating respiratory (and airway) responses with amygdala-related behaviors and amygdala-neural activities. It is essential to note, however, that with recent technological advances, future experimental studies could take the advantage of using both optogenetic and pharmacological strategies to understand the exact role that amygdalar neural circuits play on respiratory dynamics and airway function. However, to do so, it would be crucial to uncover the neuronal identity of the amygdalar pathways. Hence, another promising area of research would be performing single-neuron RNA sequencing, which is a state-of-the-art technology employed to unravel the RNA transcripts that are expressed specifically by individual neurons. Uncovering the neuronal identities would be a start point aiming to develop optogenetic and pharmacological approaches to elucidate the function of the amygdalar neural pathways in the context of behavioral-respiratory dynamics and airway function.

Moreover, the amygdala is known to play an important role in pain perception and serves as a target for opioids (Kissiwaa et al., 2020; Zhou et al., 2021). Opioid withdrawal also disrupts amygdala circuits (Gregoriou et al., 2023). However, given the potential for the amygdala to modify respiration, and the well-known deleterious effect opioids have on breathing, the potential for opioids to modify respiration through actions on the amygdala is unknown. Could the amygdala contribute to opioid-induced respiratory depression? If so, can modifying the amygdala activity be used to prevent or mitigate opioid-induced respiratory depression?

Lastly, the higher prevalence of anxiety in several airway diseases suggests that there may be a vast number of unrealized therapeutics available to improve airway health. Though the largest class of anxiolytics, the benzodiazepines, are generally not recommended in airway diseases like COPD, CF, or asthma, there are several other drug classes that may prove beneficial. Therefore, pharmacologic studies focused on the amygdala-lung axis in health and disease (both experimentally and clinically) are of high value.

## Conclusion

Respiration and airway defense are essential to life. The brainstem regions responsible for respiration and the key brainstem autonomic centers necessary for airway defense have been studied extensively for decades. However, given the observation that emotions such as fear and anxiety influence respiration and autonomic responses, regions upstream of the brainstem, such as the amygdala, have been of an area of focus for several decades as well. Surprisingly though, our understanding of the role of the amygdala in respiration and airway defense is still evolving. Given the number of pharmacologic agents and therapeutics that modulate amygdala activity/function, expanding our knowledge of the amygdala in airway physiology and respiration may reveal new airway therapeutics or shed light onto novel means to improve airway function.
